# Pharmacokinetics of Cannabis and Its Derivatives in Animals and Humans During Pregnancy and Breastfeeding

**DOI:** 10.3389/fphar.2022.919630

**Published:** 2022-07-12

**Authors:** Anaëlle Monfort, Ema Ferreira, Grégoire Leclair, Gregory Anton Lodygensky

**Affiliations:** ^1^ Platform of Biopharmacy, Faculty of Pharmacy, Université de Montréal, Montréal, QC, Canada; ^2^ CHU Sainte-Justine, Montréal, QC, Canada; ^3^ Faculty of Pharmacy, Université de Montréal, Montréal, QC, Canada; ^4^ Department of Pediatrics, Université de Montréal, Montréal, QC, Canada

**Keywords:** pregnancy, breastfeeding, pharmacokinetics, cannabis, neonates, exposure

## Abstract

Cannabis is one of the most widely used illicit drugs during pregnancy and lactation. With the recent legalization of cannabis in many countries, health professionals are increasingly exposed to pregnant and breastfeeding women who are consuming cannabis on a regular basis as a solution for depression, anxiety, nausea, and pain. Cannabis consumption during pregnancy can induce negative birth outcomes such as reduced birth weight and increased risk of prematurity and admission to the neonatal intensive care unit. Yet, limited information is available regarding the pharmacokinetics of cannabis in the fetus and newborn exposed during pregnancy and lactation. Indeed, the official recommendations regarding the use of cannabis during these two critical development periods lack robust pharmacokinetics data and make it difficult for health professionals to guide their patients. Many clinical studies are currently evaluating the effects of cannabis on the brain development and base their groups mostly on questionnaires. These studies should be associated with pharmacokinetics studies to assess correlations between the infant brain development and the exposure to cannabis during pregnancy and breastfeeding. Our project aims to review the available data on the pharmacokinetics of cannabinoids in adults, neonates, and animals. If the available literature is abundant in adult humans and animals, there is still a lack of published data on the exposure of pregnant and lactating women and neonates. However, some of the published information causes concerns on the exposure and the potential effects of cannabis on fetuses and neonates. The safety of cannabis use for non-medical purpose during pregnancy and breastfeeding needs to be further characterized with proper pharmacokinetic studies in humans feasible in regions where cannabis has been legalized. Given the available data, significant transfer occurs to the fetus and the breastfed newborn with a theoretical risk of accumulation of products known to be biologically active.

## 1 Introduction

### 1.1 Prevalence of Cannabis Use in General and Vulnerable Populations

Cannabis is the most commonly used illicit drug and its popularity is increasing with the years (Centers for Disease Control and Prevention, 2017; Statistics Canada, 2018; Statista, 2020). In Canada, 20% of people over 15—(nearly 6.2 million)– reported having used cannabis in 2020. This number represents a 6% increase in comparison to 2018 (Rotermann, 2021). In the United States and Europe, 9.6 % and 7.6% of the population over 12 years of age used it in 2017 and 2019, respectively (Dai and Richter, 2019; European Monitoring Centre for Drugs and Drug Addiction, 2022). Legalization in many countries or states and the development of new formulations (e.g., edibles and vaporizers) can explain this increase. In Canada in 2018, the number of cannabis users has increased by 5% after its legalization (Rotermann, 2021). Similarly in the United States, states where cannabis is legal exhibited the highest percentage of cannabis users between 2018 and 2019 while states where cannabis is either illegal or decriminalized showed the lowest percentage of cannabis users (Substance of Abuse and Mental Health Service Administration, Center for Behavioral Health Statistics and Quality, 2020).

Limited information is available on cannabis use during pregnancy and lactation especially since it remains illegal in many countries. However, it is well established that cannabis is the most widely used illicit drug during pregnancy. In the United States of America, a study reported that among 14,400 pregnant women, 3.8% have used cannabis during pregnancy, 6.5% were in their first trimester, 3.3% in their second trimester and 1.8% in their third trimester, while another reported an increase in use as 2.4% and 3.9% of pregnant women used cannabis in 2002 and 2014, respectively (Brown et al., 2017; Volkow et al., 2017). In Canada, Corsi et al. observed that among 732,818 pregnant women, 10,731 (1.5%) used cannabis during pregnancy with an increase from 1.2% in 2012 to 1.8% in 2017, based on interviews and medical records (Corsi et al., 2019). One study, carried out in Colorado, where cannabis is legal since 2012, showed that 5% of women used cannabis in the first weeks post-partum while breastfeeding (Crume et al., 2018). In addition, a questionnaire to clients of the Women, Infants and Children (WIC) program, a federal program in the USA for the health and nutrition of low-income pregnant and lactating women and children under 5 years old, revealed that 18% of women used cannabis at least once while breastfeeding (Wang, 2017). These values could be higher and underreported due to fear of losing their child’s custody. In Canada, no information is available to date on cannabis use during breastfeeding.

With its recent legalization, health professionals are increasingly exposed to pregnant and breastfeeding mothers who are consuming cannabis on a regular basis (Wang, 2017), as some sources on the Internet are touting cannabis as a solution for depression, anxiety, stress, pain, and nausea (Pertwee, 2012). However, when used during pregnancy, cannabis can induce negative birth outcomes such as reduced birth weight, and increased risk of prematurity and admission to the neonatal intensive care unit (Hayatbakhsh et al., 2012; Conner et al., 2016; Gunn et al., 2016). Longitudinal studies have reported behavioral and neurocognitive impairment in children exposed to cannabis *in utero* or during breastfeeding, after controlling for confounding variables such as sex of the child, ethnicity, socioeconomic status, prenatal exposure to tobacco and alcohol, or maternal substance use (Fried and Watkinson, 1990; Day et al., 1994; Goldschmidt et al., 2004; Paul et al., 2021). Moreover, cognitive deficits (e.g., executive functions, attention, memory), impulsivity and aggressive behavior were the most frequently observed effects, with long-lasting consequences up to young adulthood (Smith et al., 2006; Smith et al., 2010; Smith et al., 2016; Porath, 2018). However, most of these studies were performed in the 1980’s when THC levels in cannabis and the frequency of use were lower (Thompson et al., 2019). Therefore, it is likely that in 2022 these effects may be accentuated or that new effects may be observed. Unfortunately, there is no study available, to date, that evaluated the relationship between cannabinoid concentrations and the effects in mothers and their infants. In adults, pharmacokinetics-pharmacodynamics studies have shown that the intensity of THC effects depends on THC concentrations in the effect compartment (Grotenhermen, 2003). This relationship is likely to be similar in mothers and infants exposed to cannabis. In that regard, the Society of Obstetricians and Gynecologists of Canada has recommended in its 2022 guideline to design future studies exploring a dose-response relationship between cannabis and outcomes (Graves et al., 2022a).

### 1.2 Metabolism and Effects of Cannabinoids on Humans

Cannabis is mostly composed of cannabinoids that activate the endocannabinoid receptors (CB1 and CB2) found in the human body (Huestis, 2007). They can be classified in three types: vegetal (phytocannabinoids), endogenous (endocannabinoids) and synthetic cannabinoids (NPS). The most familiar and most studied cannabinoids are delta-9-tetrahydrocannabinol (THC), cannabidiol (CBD), cannabinol (CBN), cannabigerol (CBG), cannabichromene (CBC) and tetrahydrocannabivarin (THCV) (Health Canada and Branch, 2018).

THC is by far the most abundant and the most active cannabinoid in cannabis plants. This cannabinoid is a partial agonist of both CB1 and CB2 receptors, but has greater activity at CB1 subtypes, which is thought to account for its psychoactive effects producing changes in cognition, mood, or emotions (Mannekote Thippaiah et al., 2021). Thereby, THC is used for its psychoactive effects, causing a feeling of euphoria and well-being. Unfortunately, it is also known for its adverse effects including memory loss, tachycardia, nervousness, anxiety, paranoia and spatiotemporal disorientation. The hemp plant, also called Cannabis Sativa, is the most widely used for producing psychoactive products. It can contain up to 20% of THC depending on the part of the plant used. Manufactured products can contain from 5 to 25% of THC for marijuana joints, up to 35% for hashish, up to 60% for hashish oil and up to 90% for dabs (wax, shatter) (Gourvernement du Québec, 2021).

THC is widely distributed in adipose tissues due to its high lipophilicity. It is first metabolized in the liver by cytochromes P450 2C9, 2C19 and 3A4 to the active 11-hydroxy-delta 9-tetrahydrocannabinol (11-OH-THC), then oxidized to the inactive 11-nor-9-carboxy-delta 9-tetrahydrocannabinol (THC-COOH). Finally, THC-COOH undergoes glucuronidation by glucuronosyltransferases (UGT1A1, 1A3, 1A9 and 1A10) to become the inactive 11-nor-9-carboxy-delta 9-tetrahydrocannabinol glucuronide (THC-COOH glucuronide). THC can also be metabolized by the UDP-glucosyltransferase (UGT) in inactive delta 9-tetrahydrocannabinol glucuronide (THC glucuronide). 8-hydroxy-THC (8-OH-THC) and 8ß,11-dihydroxy-THC (8ß,11-diOH-THC), both active metabolites of THC, can also be used as biomarkers of cannabis use (Dinis-Oliveira, 2016)**.** THC metabolism is altered during pregnancy as cytochromes P450 and UGT’s activity is modified during this period. Indeed, the activity of CYP2C19, 3A4 and UGTs is increased whereas that of CYP2C9 is decreased, suggesting a different exposure to cannabinoids during pregnancy (Ward and Varner, 2019). Moreover, it is well known that both THC and CBD can be measured in breast milk, cross the placental and cross the blood-brain barrier where they can activate endogenous cannabinoid receptors given their lipophilic properties.

Cannabidiol (CBD) is the second most abundant cannabinoid in hemp plants. In contrast to THC, CBD does not have psychotropic effects and acts as a THC modulator, i.e., it attenuates the psychoactive effects of THC. This cannabinoid has lower affinity for both receptors and is a non-competitive negative allosteric modulator of the CB1 receptor. Furthermore, CBD has been shown to exhibit agonist properties on 5-HT1A receptors, potentially explaining its possible antidepressant or cognition enhancement effects (Celada et al., 2004; Russo et al., 2005). Numerous clinical studies are currently studying the benefits of CBD such as relaxing, sedative, anti-inflammatory, antispasmodic, antiepileptic, antipsychotic, antiemetic, and anti-addictive effects (Health Canada and Branch, 2018). CBD can be used with THC or alone. Indeed, many CBD oils (5%–20% CBD), pastes (30%–50% CBD), creams, lotions, patches, vaporizers and capsules do not contain any THC (Johnson, 2020). The metabolism of CBD is similar to THC, as it is first metabolized by the cytochrome P450 (CYP) to the active 7-hydroxy-cannabidiol (7-OH-CBD), then oxidized to the inactive 7-carboxy-cannabidiol (7-CBD-COOH). CBD and its metabolites are also excreted as inactive glucuronide conjugates. In the same way as THC, CBD metabolism will also be altered during pregnancy.

Cannabinol (CBN) comes from the oxidation of THC in the presence of UV rays or oxygen. It has 10% of the psychoactive effects of THC (Health Canada and Branch, 2018). Additionally, CBN could have analgesic, anti-inflammatory, antispasmodic, relaxing and anxiolytic effects. It also has adverse effects associated with a high consumption such as fatigue, weariness, and reduced heart rate (Health Canada and Branch, 2018).

Little is known concerning the minor cannabinoids CBG, CBC and THCV. CBG and CBC are present at less than 1% in Cannabis Sativa. Currently, they are being studied to determine their potential benefits and are not yet commercialized (Health Canada and Branch, 2018).

### 1.3 Objectives

The objectives of the current review are fourfold: 1. to review the literature on the main pharmacokinetics parameters of cannabinoids in animals, general population, pregnant and lactating women and neonates; 2. to identify the cannabinoids used as biomarkers of recent and past exposure to cannabis; 3. to identify the matrices that are used to assess recent and past exposure to cannabis and to assess exposure during pregnancy and lactation and; 4. to identify missing data on the pharmacokinetics of cannabinoids that should be identified as a priority in future studies aimed at monitoring fetal and neonatal toxicity.

## 2 Methods

**FIGURE 1:**
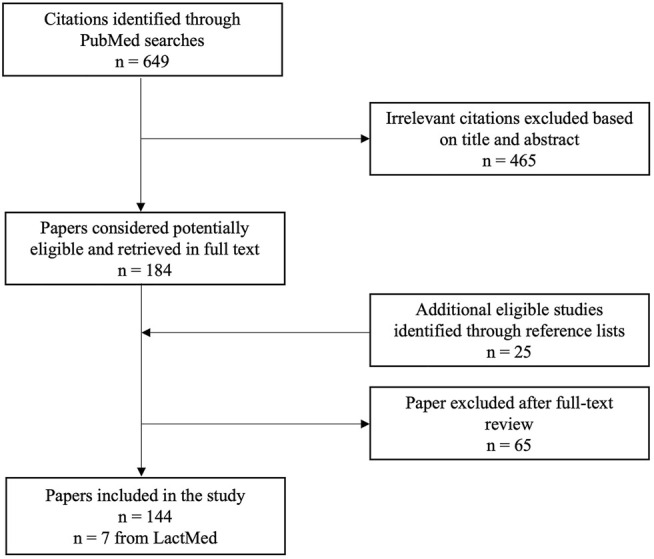
Literature review flow chart.

A literature search was conducted on PubMed and on the Drugs and Lactation Database (LactMed) to identify the literature on the pharmacokinetics of cannabinoids in animals, adults, pregnant and lactating women and neonates. The scientific literature was reviewed on the pharmacokinetics of cannabinoids in animals and humans from their inception until July 2021. The following terms were used to search the literature: cannabinoids, cannabis, THC, delta-9-THC, tetrahydrocannabinol, delta-9-tetrahydrocannabinol, cannabidiol, dronabinol, marijuana, pharmacokinetics, exposure, quantification, analysis, detection, animals, humans, adults, newborns, meconium, oral fluid, umbilical cord, hair, blood, breast milk, lactation, breastfeeding, pregnancy, and gestation. Additionally, a screening of the reference list of selected articles was performed to collect further relevant papers. Only English written articles were included in this review. Figure 1 represents the literature review flow chart.

### 2.1 Pharmacokinetics of Cannabinoids in the General Population

To assess human exposure to cannabinoids, several matrices have been characterized. Recent exposure to cannabinoids can be evaluated by quantifying cannabinoids in sweat, oral fluid, plasma, serum, and whole blood, while past exposure can be assessed by quantifying cannabinoids in hair and urine. Recent exposure is useful to determine main pharmacokinetics parameters of cannabinoids, i.e., the maximal concentration (C_max_), the time required to reach the maximal concentration (T_max_), and to understand the pathway of cannabinoids in the body. In contrast, assessing *past exposure* is useful to estimate the mean residence time of cannabinoids in the body and to assess the frequency of use of cannabis. Collecting all this information could be useful to estimate the exposure of pregnant and lactating women and their infants to cannabinoids. Different routes of administration are used in humans, each with their own C_max_, T_max_ and T_1/2_. The pharmacokinetics of cannabinoids in general population is presented in Table 1.

**TABLE 1:** Pharmacokinetics of cannabinoids in the general population.

Plasma/serum															
Administration mode	Pulmonary					Oral									Intravenous
Measured cannabinoids	THC, 11-OH-THC, THC-COOH, CBD, CBN, THC-COOH-glucuronide, THC-glucuronide					THC, 11-OH-THC, THC-COOH, CBD, 7-OH-CBD, 7-COOH-CBD									THC, 11-OH-THC, THC-COOH, THC-COOH glucuronide
Doses (mg)	3.1–8.8 (THC)	11.6–23 (THC)	26.9–36.5 (THC)	49.1–54 (THC) 58 (CBD) 1.7 (CBN)	61.2–69.4 (THC)	0.3–1.5 (THC) 1.2–1.4 (THCA) 0.7–0.9 (CBD) .8–4.4 (CBDA)	3–10.8 (THC) 5.0–10 (CBD)	14.8–16.2 (THC) 15 (CBD)	20 (THC) 20–30 (CBD)	45 (CBD) 50 (THC)	90 (THC) 90–100 (CBD)	200 (CBD)	400 (CBD)	750–800 (CBD)	2.4 to 6.5 (THC)
Cmax (ng/ml)	18.9–53 (THC) 1.4 (11-OH-THC) 10.0 (THC-COOH)	1.5–186.2 (THC) 0–11.1 (11-OH-THC) 1–103.6 (THC-COOH)	0–276 (THC) 0.7–32.3 (11-OH-THC) 7.3–310.6 (THC-COOH)	1.9–421 (THC) 0–42 (11-OH-THC) 9–207 (THC-COOH) 5.7–48.2 (CBD) 0–5.3 (CBN) 0–5.6 (THC glucuronide) 15.6–462 (TCH-COOH glucuronide)	22.5–813.2 (THC) 3–41 (11-OH-THC) 11–128 (THC-COOH)	0.4–1.01 (THC) 40.3–72.4 (THCA-A) 0.55–1.5 (11-OH-THC) 0.3–1.5 (CBD) 28.3–94.3 (CBDA) 4.3–17.1 (THC-COOH) 7.7–40.2 (THC-COOH glucuronide) 0.12–0.8 (6-OH-CBD) 151.45–159.93 (7-OH-CBD) 74.73–118.03 (7-CBD-COOH)	0.53–10.6 (THC) 0–15.0 (11-OH-THC) 1.27–281.6 (THC-COOH) 0–4.27 (CBD)	0.7–38.2 (THC) 0–19.5 (11-OH-THC) 11.0–409 (THC-COOH) 2.0–20.5 (CBD)	0.42–67.6 (THC) 0.57–38.9 (11-OH-THC) 1.9–441 (THC-COOH) 0.3–281.6 (CBD) 0.54–0.64 (CBN)	16.8–21.2 (CBD) 94.34 (THC) 66.09 (11-OH-THC) 585.57 (THC-COOH)	9–126 (CBD) 9–127.1 (THC) 20 (11-OH-THC)	148–381 (CBD) 3.19–7.56 (6-OH-CBD) 41.8–76.4 (7-OH-CBD) 221–823 (7-CBD-COOH)	114.2–181.2 (CBD)	157.1–1,050 (CBD) 180–2,800 (7-OH-CBD) 831–2,978 (7-CBD-COOH)	66–817.6 (THC), 9.1–33.0 (11-OH-THC), 36.7–84.2 (THC-COOH), 31.1–98.2 (THC-COOH glucuronide)
Tmax	1–66 min (THC), 4.2–69 min (11-OH-THC), 4.2-minutes-10.4 h (THC-COOH), 7–66 min (CBD, CBN), 13–40 min (THC glucuronide), 16.8 minutes-26 h (THC-COOH glucuronide)					0.5–12 h (THC, CBD), 0.5–24 h (11-OH-THC), 1.1–8 h (THC-COOH), 2–6 h (THC-COOH glucuronide), 0.7–6 h (6-OH-CBD), 1.5–12 h (7-OH-CBD), 2.1–24 h (7-CBD-COOH), 0.9–1.1 h (CBN), 1–2 h (THCA-A), 0.5–2 h (CBDA)									5–10 min (11-OH-THC)
															60 min (THC-COOH)
T1/2 (hours)	0.2–6.8 (THC), 0.06–4.6 (11-OH-THC), 0.18–41.9 (THC-COOH)					1.1–28.4 (THC), 2.3–41.3 (CBD), 1.0–8.8 (11-OH-THC), 9.9–113.2 (THC-COOH), 10.3–159.4 (THC-COOH glucuronide), 2.4–13.2 (6-OH-CBD), 2.9–20.2 (7-OH-CBD), 19.8–28 (7-CBD-COOH), 2–3.2 (THCA-A), 0.6–9 (CBDA)									1.6–1.9 (THC)
															5.2–6.2 days (THC-COOH)
															3.7–6.8 days (THC-COOH glucuronide)
Window of detection (hours)	>30 (THC, 11-OH-THC, THC-COOH glucuronide), >168 (THC-COOH), 4 (CBD, THC glucuronide), 3 (CBN)					>130 (THC, 11-OH-THC), >96 (CBD, 7-OH-CBD, 7-CBD-COOH), >24 (THC-COOH, CBN), 10 (6-OH-CBD)									
Articles	Ohlsson et al. (1980); Lindgren et al. (1981); Cami et al. (1991); Perez-Reyes et al. (1991); Huestis et al. (1992a); Huestis et al. (1992b); Manno et al. (2001); Huestis and Cone, (2004); Naef et al. (2004); Abrams et al. (2007); Kauert et al. (2007); Hunault et al. (2008); Skopp and Potsch, (2008); Toennes et al. (2008); Schwope et al. (2011); Stott et al. (2013a); Desrosiers et al. (2014a); Eisenberg et al. (2014); Lee et al. (2015); Marsot et al. (2016); Pichini et al. (2020a)					Ohlsson et al. (1980); Nadulski et al. (2005); Goodwin et al. (2006); Kauert et al. (2007); Skopp and Potsch, (2008); Karschner et al. (2011); Milman et al. (2011); Eichler et al. (2012); Klumpers et al. (2012); Stott et al. (2013b); Lile et al. (2013); Ahmed et al. (2014); Milman et al. (2014); Ahmed et al. (2015); Hartman et al. (2015); Manini et al. (2015); de Vries et al. (2016); Cherniakov et al. (2017a); de Vries et al. (2017); Atsmon et al. (2018a); Atsmon et al. (2018b); Birnbaum et al. (2019); Knaub et al. (2019); Patrician et al. (2019); Taylor et al. (2019); Wolowich et al. (2019); Pichini et al. (2020b); Crockett et al. (2020)									Ohlsson et al. (1980); Lindgren et al. (1981); Kelly and Jones, (1992); Naef et al. (2004); Wolowich et al. (2019)

Whole blood								
Administration mode	Pulmonary				Oral			
Measured cannabinoids	THC, 11-OH-THC, THC-COOH, THC-COOH glucuronide, THCVCOOH, THC glucuronide, THCV, CBD, CBN, CBG				THC, 11-OH-THC, THC-COOH, THC-COOH glucuronide, THCVCOOH, THC glucuronide, CBD, THCA-A, CBDA			
Doses (mg)	10–14.5 (THC)	25–33.5 (THC)	43 (THC)	50.6–54 (THC) 1.5–2 (CBD) 1.6–3.3 (CBN)	2.2–2.3 (THCA-A) 1.9–2.2 (THC) 4.4–8.8 (CBDA) 1.9–2.4 (CBD)	10 (THC)	20–25 (THC)	50–50.6 (THC) 1.5 (CBD) 3.3 (CBN)
Cmax (ng/ml)	0–71.4 (THC) 0–9.1 (11-OH-THC) 0–84.2 (THC-COOH) 0–213 (THC-COOH glucuronide)	0–210 (THC) 0–24.8 (11-OH-THC) 0–95.4 (THC-COOH) 0–259 (THC-COOH glucuronide)	8.2–192 (THC) 0.6–18 (11-OH-THC) 2.0–106 (THC-COOH)	1.3–471 (THC) 0.5–30.9 (11-OH-THC) 0.7–137 (THC-COOH) 0–10.9 (CBD) 0–37.9 (CBN) 0–1.1 (THC glucuronide) 0–248 (THC-COOH glucuronide) 1.2–3.1 (THCVCOOH) 1.0–22.7 (CBG) 1.2–4.9 (THCV)	48.9–65.4 (THCA-A) 1.4–2.3 (THC) 0.5–1.1 (11-OH-THC) 4.6–7.4 (THC-COOH) 25.8–29.3 (THC-COOH glucuronide) 55.0–74.6 (CBDA) 3.1–4.4 (CBD)	0–4 (THC) 0–4 (11-OH-THC) 6–27 (THC-COOH) 0–39 (THC-COOH glucuronide)	0–51.9 (THC) 1–20.9 (11-OH-THC) 17–253 (THC-COOH) 5–91 (THC-COOH glucuronide)	0–36.1 (THC) 2.2–23 (11-OH-THC) 12.5–152 (THC-COOH) 8–304 (THC-COOH glucuronide) 1.1–3.9 (THCVCOOH) 0.6–1.2 (THC glucuronide)
Tmax (hours)	0.5–1.9 (THC), 1.0–11 (11-OH-THC), 1.6–17.3 (THC-COOH)				1.0–5 (THC, 11-OH-THC), 1.5–5 (THC-COOH), 1.5–8 (THC-COOH glucuronide), 0.6–1 (CBD), 1.0–3.5 (THCVCOOH), 1.5–3.5 (THC glucuronide), 1.2–1.3 (THCA-A), 0.8–1.1 (CBDA)			
T1/2 (hours)	0.2–6.8 (THC), 0.06–4.6 (11-OH-THC), 0.18–41.9 (THC-COOH)				1.6–1.9 (THC), 2.2–2.5 (11-OH-THC), 3.9–5.2 (THC-COOH), 19.8–23.3 (THC-COOH glucuronide), 0.5–0.9 (CBD), 5.3 (THCA-A), 0.7–0.8 (CBDA)			
Window of detection (hours)	>72 (THC, 11-OH-THC, THC-COOH, THC-COOH glucuronide), 44 (THCVCOOH), 0.5 (CBD), 2.1 (CBN), 0.6 (THC glucuronide), 0.5 (CBG), 0.17 (THCV)				>72 (THC, 11-OH-THC, THC-COOH, THC-COOH glucuronide), 26 (THCVCOOH), 24 (THCA-A), 8 (CBDA), 6 (CBD), 5 (THC glucuronide)			
Articles	Schwope et al. (2011); Schwope et al. (2012); Fabritius et al. (2013); Desrosiers et al. (2014a); Hartman et al. (2015); Newmeyer et al. (2016); Hartley et al. (2019); Spindle et al. (2019)				Karschner et al. (2012); Newmeyer et al. (2016); Newmeyer et al. (2017); Pellesi et al. (2018); Spindle et al. (2020a)			

Urine											
Administration mode	Pulmonary					Oral					Intravenous
Measured cannabinoids	CBD, THC-COOH, THC, 11-OH-THC, THC-glucuronide, THC-COOH glucuronide, 8.11-diOH-THC, THCV, 8-OH-THC, CBN					THC, 11-OH-THC, THC-COOH, THC-COOH glucuronide, THCVCOOH, THC glucuronide, CBD, THCA-A, CBDA					THC-COOH, THC-COOH glucuronide
Doses (mg)	5.8 (CBD)	15.2–20 (THC)	26.9–33.8 (THC)	54 (THC)	100 (CBD) 3.7 (THC)	0.7–0.9(CBD) 2.8–4.4 (CBDA) 0.3–1.0 (THC) 1.2–1.4 (THCA-A)	45 (CBD)	90–100 (CBD)	400 (CBD)	800 (CBD)	5 (THC)
Cmax (ng/ml)	6.0–15.1 (CBD)	0–21.5 (THC), 0–79.3 (11-OH-THC), 0–156.7 ng/ml or 34.6–171.6 µg (THC-COOH)	0–53.3 (THC) 0–169.0 (11-OH-THC), 0–472.2 ng/ml or 305 µg (THC-COOH) 92.3 (8 β-di-OH-THC)	0–36.6 (THC-COOH) 1.3–45.2 (THC glucuronide) 8.7–2080 (THC-COOH glucuronide)	15.3–631.1 (CBD), 0–29.9 (THC-COOH)	239.1–242.9 µg (7-OH-CBD) 42.7–65.9 µg (7-CBD-COOH) 7.6–8.7 µg (6-β-OH-CBD) 0.7–2.5 µg (6-α-OH-CBD) 0.08–0.55 µg (THC glucuronide) 0.24–0.80 µg (THC-COOH) 35.98–217.02 µg (THC-COOH glucuronide)	0.7–1.2 (CBD)	1.4–2941 (CBD) 0–2.9 (THC-COOH)	2.9–4.6 (CBD conjugates)	2.8–3.7 (CBD conjugates)	1.0–2.8 (THC-COOH) 55.5–96.6 (THC-COOH glucuronide)
Tmax (hours)	2 (THC), 3 (11-OH-THC), 6 (THC glucuronide), 3–96 (THC-COOH), 6–12 (THC-COOH glucuronide), 0.5–5 (CBD)					1.0–5 (THC, 11-OH-THC), 1.5–5 (THC-COOH), 1.5–8 (THC-COOH glucuronide), 0.6–1 (CBD), 1.0–3.5 (THCVCOOH), 1.5–3.5 (THC glucuronide), 1.2–1.3 (THCA-A), 0.8–1.1 (CBDA)					
T1/2 (hours)	0.2–6.8 (THC), 0.06–4.6 (11-OH-THC), 0.18–41.9 (THC-COOH)					1.6–1.9 (THC), 2.2–2.5 (11-OH-THC), 3.9–5.2 (THC-COOH), 19.8–23.3 (THC-COOH glucuronide), 0.5–0.9 (CBD), 5.3 (THCA-A), 0.7–0.8 (CBDA)					1.6–2 days (THC-COOH glucuronide)
Window of detection (hours)	14 days (THC-COOH), 97 (CBD), >30 (THC glucuronide, THC-COOH glucuronide), 8 (11-OH-THC), 7 (THC)					>72 (THC, 11-OH-THC, THC-COOH, THC-COOH glucuronide), 26 (THCVCOOH), 24 (THCA-A), 8 (CBDA), 6 (CBD), 5 (THC glucuronide)					12 days (THC-COOH, THC-COOH glucuronide)
Articles	Huestis and Cone, (1998); Manno et al. (2001); Westin et al. (2009); Marsot et al. (2016); Pacifici et al. (2018); Spindle et al. (2020b)					Karschner et al. (2012); Newmeyer et al. (2016); Newmeyer et al. (2017); Pellesi et al. (2018); Spindle et al. (2020a)					Kelly and Jones, (1992)

	**Oral fluid**									**Sweat**	**Hair**
**Administration mode**	**Pulmonary**					**Oral**				**Oral**	**Pulmonary**
Measured cannabinoids	THC, THC-COOH, THC-A, CBD, CBN					THC, 11-OH-THC, THC-COOH, THC-COOH glucuronide, THC glucuronide, CBD, CBN, CBG, THCV, THCA-A, CBDA				THC, 11-OH-THC, THC-COOH, THC-COOH glucuronide, THC glucuronide, CBD, CBN, THCA-A, CBDA	THC, CBD, CBN, THC-COOH, 11-OH-THC
Doses (mg)	1.6 (THC) 58 (CBD)	10 (THC)	18–25.6 (THC)	33–43 (THC)	54 (THC) 2 (CBD) 1.7 (CBN)	1 (THC) 1 (CBD)	10 (THC)	20–25 (THC)	50 (THC) 1.5(CBD) 3.3 (CBN)	0.1-unknown (THC) 0.9 (CBD) 1.5 (THCA-A) 2.8 (CBDA)	unknown
Cmax (ng/ml)	0–21.5 (THC) 0–267.4 (CBD)	0–1,063 (THC) 0–1.1 (THC-COOH)	55.4–123,120 (THC) 0–0.1 (THC-COOH)	19–71,747 (THC) 0.1–2.4 (THC-COOH) 4.8–2,910 (THCA-A) 6.0–756 (CBN)	68–10,284 (THC) 0.02–0.8 (THC-COOH) 2,6–588 (CBD) 4,8–1,558 (CBN)	0.2–1.2 (THC) 1.0–6.9 (THCA-A) 0.6–1.6 (CBD) 14.3–202 (CBDA)	11–414 (THC) 0–0.5 (THC-COOH)	0–667 (THC) 0–1,087 (THC-COOH) 0.5–2.1 (CBD) 1.3–13.0 (CBN)	39.3–2,111 (THC) 0.2–1,281 (THC-COOH) 0.2–1.2 (11-OH-THC) 1.3–19.4 (THCV) 2.0–29.7 (CBD) 3.5–90.1 (CBG)	0.3–152 ng/patch (THC) 0.4–1 ng/patch (CBD) 0.4–0.5 ng/patch (CBN)	0.02–4.0 ng/mg (THC) 0.01–0.5 ng/mg (CBD) 0.003–0.7 ng/mg (CBN) 0.004–0.08 ng/mg (THC-COOH) 0.001–0.004 ng/mg (11-OH-THC)
Tmax (hours)	0.02–3.5 (THC), 0.3–6 (THC-COOH), 0.25–0.5 (CBD), 0.2–0.6 (CBN), 3.5 (THCA-A)					10.2–24 (THC), 0.3–1.5 (11-OH-THC), 2–8 (THC-COOH), 0.3–4 (CBD), 0.3 (CBG, THCV), 0.5–4 (THCA-A), 0.5–2 (CBDA)				>10	
T1/2 (hours)	0.02–18.9 (THC), 0.8–7.2 (THC-COOH), 0.4–2.7 (CBN), 0.3–4.4 (THCA-A)					0.5–42 (THC), 2.8–4.5 (CBD), 1.3–18 (THCA-A), 3.1–5.4 (CBDA)					
Window of detection (hours)	>48 (THC), >22 (THC-COOH), 18 (CBD), 6 (CBN)					>48 (THC, THC-COOH), 3.5 (11-OH-THC), 11 (CBD, CBN), 14 (CBG), 3.5 (THCV), 6 (THCA-A, CBDA)				4 weeks	3 months
Articles	Huestis and Cone, (2004); Moore et al. (2006); Kauert et al. (2007); Milman et al. (2010); Toennes et al. (2010); Lee et al. (2012); Fabritius et al. (2013); Toennes et al. (2013); Marsot et al. (2016); Pacifici et al. (2018); Spindle et al. (2019); Pichini et al. (2020b)					Moore et al. (2006); Milman et al. (2011); Newmeyer et al. (2017); Spindle et al. (2020a); Pichini et al. (2020b)				Kintz et al. (2000); Huestis et al. (2008); Gambelunghe et al. (2016); Pichini et al. (2020b); Spindle et al. (2020b)	Gambelunghe et al. (2016); Taylor et al. (2017); Tzatzarakis et al. (2017); Hartley et al. (2019)

#### 2.1.1 Recent exposure to Cannabis

##### 2.1.1.1 Pharmacokinetics of Cannabinoids in Plasma and Serum

Plasma and serum matrices have been characterized in many clinical studies as the drug’s pharmacodynamic and toxicity is related to concentrations in these matrices (Ohlsson et al., 1980; Lindgren et al., 1981; Cami et al., 1991; Perez-Reyes et al., 1991; Huestis et al., 1992a; Huestis et al., 1992b; Kelly and Jones, 1992; Manno et al., 2001; Huestis and Cone, 2004; Naef et al., 2004; Nadulski et al., 2005; Goodwin et al., 2006; Abrams et al., 2007; Kauert et al., 2007; Hunault et al., 2008; Skopp and Potsch, 2008; Toennes et al., 2008; Schwilke et al., 2009; Karschner et al., 2011; Milman et al., 2011; Schwope et al., 2011; Eichler et al., 2012; Klumpers et al., 2012; Stott et al., 2013a; Stott et al., 2013b; Lile et al., 2013; Desrosiers et al., 2014a; Ahmed et al., 2014; Eisenberg et al., 2014; Milman et al., 2014; Ahmed et al., 2015; Hartman et al., 2015; Lee et al., 2015; Manini et al., 2015; de Vries et al., 2016; Marsot et al., 2016; Cherniakov et al., 2017a; de Vries et al., 2017; Atsmon et al., 2018a; Atsmon et al., 2018b; Birnbaum et al., 2019; Knaub et al., 2019; Patrician et al., 2019; Taylor et al., 2019; Wolowich et al., 2019; Pichini et al., 2020a; Pichini et al., 2020b; Crockett et al., 2020; Hobbs et al., 2020). Cannabinoids tested, routes of administration, times of detection and pharmacokinetics parameters (C_max_, T_max_, T1/2) are all reported in Table 1. The variation in concentrations after pulmonary administration can be explained by multiple factors including first the difference in dosages but also, the difference in formulations, the fasting/fed stage, the addition of alcohol, tobacco, or other drugs and the interindividual variability regarding the age, the smoking topography, and the history of cannabis use. Variation of T_max_ between studies is mostly caused by the variation in sampling times. Skopp observed that THC-COOH was detectable up to 48 h and THC-COOH glucuronide for more than 48 h in plasma samples of light, moderate and heavy smokers (Skopp and Pötsch, 2008).

Oral formulations were composed of THC alone, THC + CBD or CBD alone (Ohlsson et al., 1980; Nadulski et al., 2005; Goodwin et al., 2006; Schwilke et al., 2009; Karschner et al., 2011; Milman et al., 2011; Eichler et al., 2012; Klumpers et al., 2012; Stott et al., 2013b; Lile et al., 2013; Ahmed et al., 2014; Milman et al., 2014; Ahmed et al., 2015; Manini et al., 2015; de Vries et al., 2016; de Vries et al., 2017; Atsmon et al., 2018a; Atsmon et al., 2018b; Birnbaum et al., 2019; Knaub et al., 2019; Patrician et al., 2019; Taylor et al., 2019; Wolowich et al., 2019; Pichini et al., 2020b; Crockett et al., 2020; Hobbs et al., 2020; Pérez-Acevedo et al., 2020; Pérez-Acevedo et al., 2021). A high interindividual variability was observed across studies, explaining the large intervals of concentration. The fasting/fed stage, the formulation, the use of alcohol, the age and the dosage also influenced cannabinoid concentrations. As expected, reported T_max_ were longer after oral than after pulmonary consumption. The high variation between half-lives is explained by the difference in sampling times between studies. Indeed, sampling periods vary between 2 and 130 h.

THC, CBD and THC metabolites (11-OH-THC and THC-COOH) are well characterized in plasma and serum and have demonstrated to be crucial in understanding the effects of cannabis in humans. Interestingly, THC-COOH known to be inactive, is eliminated more slowly and has higher plasma concentrations than THC. Thus, this metabolite is useful for assessing cannabis exposure over a longer period. In addition, ratios of THC on its metabolites or on CBD is promising to assess recent exposure to cannabis (Pichini et al., 2020b). THC-COOH glucuronide has an even longer half-life than THC-COOH and is detectable in most of the plasma samples, 48 h after the last consumption of cannabis (Skopp and Pötsch, 2008). Therefore, it could be used to determine someone’s last cannabis consumption. CBD metabolites, 7-OH-CBD and 7-CBD-COOH are measured at higher concentrations than CBD. Thus, they should be included in pharmacokinetics studies after CBD consumption. Studies evaluating the effects of cannabis on humans should also quantify CBN as it can evaluate recent cannabis use. In plasma and serum, sampling should be performed for at least 24 h with more samples during the first hour after pulmonary administration controlling for THC, 11-OH-THC, THC-COOH, THC-COOH glucuronide, CBD, 7-OH-CBD, 7-CBD-COOH and CBN. Finally, attention should be paid to the use of food, alcohol, tobacco, or drugs before the beginning of the study as it could influence the pharmacokinetics of cannabinoids in plasma.

##### 2.1.1.2 Pharmacokinetics of Cannabinoids in Whole Blood

Whole blood gives similar information than plasma and serum. This matrix is advantageous as it does not need to be centrifuged immediately after extraction. However, it is less stable than plasma or serum and samples should be analyzed quickly after their collection.

THC, 11-OH-THC, THC-COOH, THC-COOH glucuronide, THCVCOOH, THC glucuronide, THCV, CBD, CBN and CBG were measured in whole blood (Table 1) (Schwope et al., 2011; Karschner et al., 2012; Schwope et al., 2012; Fabritius et al., 2013; Desrosiers et al., 2014a; Hartman et al., 2015; Newmeyer et al., 2016; Newmeyer et al., 2017; Pellesi et al., 2018; Hartley et al., 2019; Spindle et al., 2019; Spindle et al., 2020a). Pharmacokinetics of THC, 11-OH-THC, THC-COOH, THC-COOH glucuronide, CBD and CBN seem to be similar in plasma, serum, and whole blood. Therefore, the same considerations should be applied to pharmacokinetics studies in whole blood.

##### 2.1.1.3 Pharmacokinetics of Cannabinoids in Oral Fluid (Saliva)

Oral fluid has many advantages for cannabinoid quantification: its collection is simple and non-invasive. Moreover, it gives a good idea of someone’s recent exposure to cannabis. Indeed, oral fluid is often used to detect drivers under the influence of cannabis. However, one should be careful of contamination of the oral cavity after smoking cannabis. As THC-COOH concentrations is the result of THC hepatic metabolism and is not present in cannabis smoke, its quantification is highly important in oral fluid. THC, 11-OH-THC, THC-COOH, THC-COOH glucuronide, THC glucuronide, CBD, CBN, CBG, THCV, THCA-A and CBDA were quantified in oral fluid (Huestis and Cone, 2004; Moore et al., 2006; Kauert et al., 2007; Milman et al., 2010; Toennes et al., 2010; Milman et al., 2011; Lee et al., 2012; Fabritius et al., 2013; Toennes et al., 2013; Marsot et al., 2016; Newmeyer et al., 2017; Pacifici et al., 2018; Spindle et al., 2019; Pichini et al., 2020a; Spindle et al., 2020a; Pérez-Acevedo et al., 2021). Pharmacokinetics parameters of cannabinoids in oral fluid are presented in Table 1. High variability in concentrations after pulmonary administration can be explained by the smoking topography (puff volume, duration, flow), cannabis use history, and the contamination of the oral cavity, in addition to the difference in doses. THC, THC-COOH, CBD, CBD-COOH and CBN should be quantified in oral fluid for pharmacokinetics studies or to assess the short-term exposure to cannabis. Finally, pharmacokinetics studies using oral fluid should collect samples for at least 24 h starting at baseline.

##### 2.1.1.4 Pharmacokinetics of Cannabinoids in Sweat

Pichini, Perez-Acevedo, Gambelunghe, Huestis and Kintz have assessed the exposure to cannabis by quantifying cannabinoids in sweat as a replacement of oral fluid (Kintz et al., 2000; Huestis et al., 2008; Gambelunghe et al., 2016; Pichini et al., 2020b; Pérez-Acevedo et al., 2021). THC, 11-OH-THC, THC-COOH, THC-COOH glucuronide, THC glucuronide, CBD, CBN, THCA-A and CBDA were the cannabinoids targeted in these studies. C_max_, T_max_ and T_1/2_ are presented in Table 1. After oral doses of cannabis, only THC, CBD and CBN were detected in sweat. Sweat can be employed for monitoring drug users in the workplace, treatment, and judicial program (Huestis et al., 2008). If sweat is chosen to assess the exposure to cannabis, THC, CBD and CBN should be monitored. Further studies are needed to better understand the pharmacokinetics of these cannabinoids in sweat.

#### 2.1.2 Past Exposure

##### 2.1.2.1 Pharmacokinetics of Cannabinoids in Urine

To assess the past exposure to cannabis, urine is the ideal matrix as cannabinoids are detectable for many weeks. Multiple studies have measured THC and its metabolites, THCV, CBD and its metabolites, CBN, THCA-A and CBDA concentrations in urine (Table 1) (Kelly and Jones, 1992; Huestis and Cone, 1998; Manno et al., 2001; Westin et al., 2009; Desrosiers et al., 2014b; Manini et al., 2015; Marsot et al., 2016; Pacifici et al., 2018; Patrician et al., 2019; Pichini et al., 2020b; Spindle et al., 2020b; Pérez-Acevedo et al., 2020; Pérez-Acevedo et al., 2021)**.** Only THC, 11-OH-THC, THC-COOH, THC-COOH glucuronide, THC glucuronide and CBD were detected after pulmonary administration (Huestis and Cone, 1998; Manno et al., 2001; Desrosiers et al., 2014b; Marsot et al., 2016; Pacifici et al., 2018; Spindle et al., 2020b). THC-COOH was detected in urine up to 14 weeks after the last consumption. If urine is used, THC, 11-OH-THC, THC-COOH, THC-COOH, THC glucuronide, CBD, 7-OH-CBD and 7-CBD-COOH should be tested.

##### 2.1.2.2 Pharmacokinetics of Cannabinoids in Hair

Past exposure to cannabis can also be detected in hair samples. Hartley, Gambelunghe, Taylor and Tzatzarakis have quantified THC, 11-OH-THC, THC-COOH, CBD and CBN in hair samples (Table 1) (Gambelunghe et al., 2016; Taylor et al., 2017; Tzatzarakis et al., 2017; Hartley et al., 2019). This matrix enables the detection of cannabis consumption up to 7 months prior sampling. THC-COOH quantification in hair samples is helpful to distinguish cannabis consumption from passive exposure (Gambelunghe et al., 2016). Thus, THC, CBD, CBN and THC-COOH should be tested to assess cannabis exposure in hair samples. However, more studies are needed to understand the pharmacokinetics of cannabinoids in hair. Indeed, it is, yet, not possible to make correlations between the presence of any cannabinoid, their concentration, and the last time of cannabis use.

### 2.2 Pharmacokinetics of Cannabinoids in Pregnant or Lactating Women and Newborns

Assessing the exposure of newborns to cannabis during pregnancy and lactation is essential to understand the effects of cannabis on the newborn’s development. Unfortunately, only a few studies have attempted to evaluate cannabis exposure during these two periods. Indeed, most of published studies aimed to assess whether the newborn was exposed to cannabis during pregnancy and, thus, used qualitative analysis rather than quantitative analysis. Moreover, no study has assessed the pharmacokinetics of cannabinoids in pregnant women, even though the pharmacokinetics will be altered during pregnancy and could influence the effects of cannabis on women. Therefore, pharmacokinetics studies in pregnant women could bring crucial information for the mother and her newborn, as no non-invasive method exists to quantify cannabinoids in fetuses. Indeed, exposition during pregnancy was only evaluated in one study testing urine samples of a mother and shortly after birth in meconium, hair, urine, cord blood and umbilical cord samples of newborns. Newborn’s exposure during breastfeeding has been assessed in some studies by quantifying cannabinoids in breast milk samples. Cannabinoid concentrations in pregnant and lactating women and neonates are summarized in Table 2.

**TABLE 2:** Pharmacokinetics of cannabinoids in pregnant and lactating women and neonates.

Matrices	Measured cannabinoids	Time of sampling	Measured concentrations (ng/ml)	Articles
Pregnant woman urine	THC-COOH	Up to 14 weeks after the last cannabis consumption	3.9–348.1 ng/ml	Kintz et al.(2000)
Breast milk	THC, THC-COOH, 11-OH-THC, CBG, THC-COOH glucuronide, THC-glucuronide, CBD, CBC, CBN, THCV, THCVCOOH	>6 weeks after the last cannabis consumption	Breast milk: 1.0–420 (THC), 1.1–12.8 (11-OH-THC), 0.8–11 (THC-COOH), 0.4–17 (CBD), 0.4–7.1 (CBG) Maternal plasma: 7.2 (THC), 2.5 (11-OH-THC), 19 (THC-COOH) Infant feces: 347 (THC), 67 (11-OH-THC), 611 (THC-COOH)	Huestis and Cone, (1998); Taylor et al. (2017); Tzatzarakis et al. (2017); Grant et al. (2018); Presence of Δ9, (1982); Marchei et al. (2011); Baker et al. (2018); Bertrand et al. (2018)
Meconium	THC, THC-COOH, 11-OH-THC, CBD, CBN, diOH-THC THC-COOH glucuronide, 8-OH-THC	0–48 h after delivery	THC: 2.4–81.2 ng/g THC-COOH: 1.4–1,856 ng/g 11-OH-THC: 1.2–929 ng/g diOH-THC: 2.4–887.4 ng/g CBD: 7.1–335.3 ng/g CBN: 10.1–189.8 ng/g THC-COOH glucuronide: 19.4–306.8 ng/g 8-OH-THC: 7.9–16.7 ng/g	Moore et al. (1996); ElSohly and Feng, (1998); Feng et al. (2000); Boskovic et al. (2001); Vinner et al. (2003); Coles et al. (2005); Marchei et al. (2006); Montgomery et al. (2006); Gray et al. (2009); Gray et al. (2010a); Tynon et al. (2015); Colby, (2017); Lamy et al. (2017); Palmer et al. (2017); Prego-Meleiro et al. (2017); Mantovani et al. (2018); Sempio et al. (2020); Moss et al. (2021); Wymore et al. (2021)
Umbilical cord	THC, THC-COOH, 11-OH-THC, CBD, THC-COOH glucuronide diOH-THC, THC glucuronide	Immediately after delivery	THC-COOH: 0.2–20.9 ng/g THC: 0.2–1.3 ng/g 11-OH-THC: 0.3–3.1 ng/g	ElSohly and Feng, (1998); Gray et al. (2010a); Kim et al. (2018); Wymore et al. (2021)
Cord blood	THC, THC-COOH		Maternal blood: <0.2–6 ng/ml (THC), 2.3–225 ng/ml (THC-COOH) Cord blood: <0.2–1.0 ng/ml (THC), 0.4–18 ng/ml (THC-COOH)	Ostrea et al.(2001)
Urine	THC-COOH	48 h after delivery	Maternal urine: 10–685 μg/g of creatinine Infant urine: 2.7–37.5 μg/g of creatinine	Ostrea et al.(2001)
Hair (neonates)	THC, CBN	12–24 h after delivery	THC: 0.22–0.86 ng/mg CBN: <0.2–0.25 ng/mg Cannabinoids: 1.9 ng/mg	ElSohly and Feng, (1998); Gray et al. (2010b); Wu et al. (2018); Wymore et al. (2021)

#### 2.2.1 Pharmacokinetics of Cannabinoids in Pregnant Women

Measuring cannabinoid concentrations in pregnant women is the first step in understanding the exposure of newborns during this period. Indeed, a drug pharmacokinetics can be altered due to major physiological changes in women during this period. Unfortunately, no study has measured plasmatic concentrations of cannabinoids. Westin et al. (2009) measured the concentration of THC-COOH in the urine of one pregnant woman during 14 weeks after the last consumption of cannabis reported by the woman. Concentration at week 2 was 346 ng/ml, and concentration at week 14 was 3.9 ng/ml. Assuming that the mother’s report is accurate, THC-COOH could be detected in urine up to 14 weeks after last consumption. As the THC dose taken by the pregnant mother in this study is unknown, it is difficult to compare THC-COOH concentrations to the ones obtained in the general population. For cannabis, it is expected that THC clearance will be increased due to a higher activity of the CYP2C19 and UGTs, enzymes responsible of THC metabolism (Grant et al., 2018). Moreover, an increase of the body fat could increase the volume of distribution of THC. Thus, THC concentrations might be decreased, and THC metabolite concentrations might be increased. However, free cannabinoid concentrations could increase, as THC and THC metabolites are highly bound to serum proteins and albumin which are decreased during pregnancy. During the third trimester, these three cannabinoids could accumulate in the fetus due to a higher concentration of fetal serum proteins than maternal serum proteins and a lower pH of fetal blood than maternal blood (Ward and Varner, 2019).

This could be confirmed in future studies in pregnant women by measuring THC, 11-OH-THC, THC-COOH, CBD, OH-CBD, CBD-COOH and CBN concentrations in blood for at least 24 h at each trimester while controlling the amount of cannabis ingested by the mother.

#### 2.2.2 Pharmacokinetics of Cannabinoids in Breastfeeding Women

Breast milk can be tested in adults to assess short-term exposure to cannabis. It has the advantage of assessing both the exposure of the mother and the breastfed child to cannabis. Unfortunately, only a few studies have measured cannabinoids in breast milk and assessed their pharmacokinetics in this matrix (Presence of Δ9, 1982; Marchei et al., 2011; Baker et al., 2018; Bertrand et al., 2018; Moss et al., 2021; Wymore et al., 2021; Sempio et al., 2020)**.** Indeed, such studies are difficult to conduct because many mothers refuse to enroll by fear of being judged or underreport their cannabis consumption making it difficult to correlate measured concentrations and the dose taken or simply stop their consumption during the study. Table 1 summarizes the pharmacokinetics parameters of cannabinoids in breast milk. Baker was the only one to assess the transfer of cannabis into breast milk by measuring cannabinoids at specific time points (Baker et al., 2018). If 11-OH-THC and THC-COOH were not quantifiable in any of the breast milk samples, THC was still quantifiable 4 hours after the cannabis administration suggesting that pharmacokinetic studies in breast milk should be performed over a longer period. Indeed, THC metabolites were probably not detected in breast milk because of the short sampling period. Bertrand et al. measured concentrations for THC, 11-OH-THC and CBD but did not detect cannabinol in any of the samples (Bertrand et al., 2018). Most concentrations were measured within 48 h after the last consumption, but one sample had THC concentrations up to 6 days after the last cannabis intake. Moss et al. (Moss et al., 2021) measured similar cannabinoid concentrations (THC, 11-OH-THC, THC-COOH and CBD) in breast milk after inhalation of cannabis or after edible use within 40 h after last consumption. In both cases, THC and CBD concentrations were at least two times higher in breast milk than in plasma. Inversely, THC metabolite concentrations were at least two times higher in plasma than in breast milk. These results are expected knowing the lipophilicity of THC and CBD. THC was detectable up to 53 h after the last cannabis use. Finally, Sempio et al. found THC, 11-OH-THC, THC-COOH, CBD and CBG detectable concentrations in 36 samples collected at an unknown time after the last cannabis consumption (Sempio et al., 2020). Other cannabinoids were not detected in any of the samples.

Overall, cannabinoid concentrations in breast milk are consistent from one study to another. THC, 11-OH-THC, THC-COOH, CBD and CBG will transfer into breast milk; therefore, their pharmacokinetics should be assessed in this matrix for at least 24 h to assess neonates’ exposure to these cannabinoids.

#### 2.2.3 Pharmacokinetics of Cannabinoids in Newborns

##### 2.2.3.1 Pharmacokinetics of Cannabinoids in Meconium

Meconium is by far the most tested matrix to assess the exposure of newborns to cannabis, as it is easy to collect and non-invasive. Meconium samples are collected in diapers during the first 48 h after delivery, until the formation of a milk stool. Seventeen studies have measured cannabinoids in meconium (Moore et al., 1996; ElSohly and Feng, 1998; Feng et al., 2000; Boskovic et al., 2001; Ostrea et al., 2001; Vinner et al., 2003; Coles et al., 2005; Marchei et al., 2006; Montgomery et al., 2006; Gray et al., 2009; Gray et al., 2010a; Gray et al., 2010b; Tynon et al., 2015; Colby, 2017; Lamy et al., 2017; Palmer et al., 2017; Prego-Meleiro et al., 2017; Kim et al., 2018; Mantovani et al., 2018). All studies lacked information on the dose taken by the mother and only two studies reported the last trimester of cannabis use (Gray et al., 2009; Gray et al., 2010a). Cannabinoids were mainly quantifiable when the mother used cannabis during the third trimester. Gray et al. observed that cannabinoids were detectable in 7% of samples when the mother stopped her consumption in the first trimester, in 11% of samples when the mother stopped in the second trimester and in 51% of samples when the mother stopped during the third trimester (Gray et al., 2009; Gray et al., 2010a). No correlation was established between the presence of certain cannabinoids and the trimester at which cannabis use was stopped. However, according to Gray’s results, diOH-THC and 11-OH-THC were only detected when cannabis was last used during the third trimester, CBN was detected if the last use was during the second and third trimesters and THC-COOH was detected regardless of when it was used during pregnancy. (Gray et al., 2010a). THC-COOH concentrations appear to be lower when the mother has stopped using cannabis in the 1st trimester of pregnancy rather than in the 3rd trimester. Gray and Ostrea observed that maternal self-report was more sensitive than meconium in detecting neonatal exposure to cannabis (Gray et al., 2009; Gray et al., 2010b). However, in both studies, only THC-COOH was quantified in meconium, suggesting that other cannabinoids should be used to assess the newborn’s exposure to cannabis. Indeed, Marchei, Feng and Coles observed that THC, 11-OH-THC, and diOH-THC were present in meconium samples without THC-COOH, increasing the number of positive meconium samples (ElSohly and Feng, 1998; Feng et al., 2000; Coles et al., 2005; Marchei et al., 2006).

All these results suggest that pharmacokinetics studies including meconium samples should monitor at least THC, 11-OH-THC, THC-COOH, diOH-THC, CBD, 7-OH-CBD, 7-CBD-COOH, CBN and glucuronide cannabinoids and control for the type of consumption of the mother during pregnancy as well as the last trimester of cannabis use.

##### 2.2.3.2 Pharmacokinetics of Cannabinoids in the Umbilical Cord and Cord Blood

The umbilical cord is the second most used matrix to assess cannabis exposure during pregnancy as it gives similar results than meconium (Montgomery et al., 2006; Colby, 2017; Kim et al., 2018; Wu et al., 2018). It has the advantages over meconium that 1- it can be tested immediately after delivery, 2- it is easier to collect and 3- it is always available. Colby and Montgomery observed 76% and 90.7% of agreement between umbilical cord and meconium cannabis concentrations suggesting that umbilical cord can be used as a replacement of meconium for assessing exposure during the third trimester (Montgomery et al., 2006; Colby, 2017). However, results are very scarce, and few cannabinoids have been tested in the umbilical cord. Further studies should be conducted for the same reasons as meconium. Cord blood can also be used at delivery to quantify THC and THC-COOH up to 26 h after smoking (Blackard and Tennes, 1984).

##### 2.2.3.3 Pharmacokinetics of Cannabinoids in Neonatal Hair

Neonatal hair was tested by Boskovic, Vinner, Kintz and Bar-Oz (Boskovic et al., 2001; Bar-Oz et al., 2003; Vinner et al., 2003; Kintz, 2015). It can be used to assess the newborn exposure during the third trimester**.** Bar-Oz observed a sensitivity of 98% in meconium and 71% in neonatal hair for cannabinoids suggesting a better performance using meconium samples (Bar-Oz et al., 2003). Information is very scarce on the quantification of cannabinoids in neonatal hair. Therefore, it is not yet determined if the use of neonatal hair would be adequate to assess cannabis exposure during pregnancy.

##### 2.2.3.4 Pharmacokinetics of Cannabinoids in Urine

Urine was tested once by Vinner, 48 h after delivery (Vinner et al., 2003). Urine samples can be used at delivery to assess cannabis consumption by the mother 1–8 days before delivery.

##### 2.2.3.5 Pharmacokinetics of Cannabinoids in Newborns Exposed During Breastfeeding

Cannabinoids were only quantified once in child urine and feces after cannabis exposure during breastfeeding. Perez-Reyes measured THC, 11-OH-THC and THC-COOH concentrations in child feces while no cannabinoids were detected in child urine (Presence of Δ9, 1982)**.** The results obtained in this study suggest that the THC present in breast milk was absorbed and metabolized by the infant because the proportion of 11-OH-THC and THC-COOH to the parent was much higher in the feces than in breast milk.

#### 2.2.4 Summary of the Knowledge on the Cannabinoid's Pharmacokinetics in Pregnant and Lactating Women and Neonates

There is a lack of studies that assessed the exposure of women and neonates to cannabis during pregnancy and lactation. Yet, to understand the effect of cannabis on the newborn’s development, it is first important to assess their exposure during these two periods. Quantifying cannabinoids in pregnant women’s blood will first help to understand how the pharmacokinetic parameters such as the C_max_, the T_max_ or the clearance are altered during pregnancy and then to assess the fetus exposure to cannabis. Infant blood would be the best matrix to assess the short-term exposure of neonates to cannabinoids during the last days of pregnancy or during lactation. Meconium and umbilical cord can be used as non-invasive matrices to assess the exposure during pregnancy, but meconium cannot be used for the first weeks of gestation and umbilical cord can only be used for the third trimester. For cannabis exposure during lactation, breast milk, urine and feces would be the best non-invasive indicator for short-term and long-term exposure to cannabis.

Based on available literature in humans we believe that THC, 11-OH-THC, THC-COOH, diOH-THC, THC-COOH glucuronide, CBD, 7-OH-CBD, 7-CBD-COOH, and CBN are of importance as they cross the placenta, can be found in breast milk or will be produced by the newborn’s metabolism and should be monitored to assess the exposure of fetuses and neonates to cannabis during pregnancy and lactation.

### 2.3 Pharmacokinetics of Cannabinoids in Animals

Preclinical studies on animals are used to assess the efficacy and toxicity of new drugs or formulations before the administration in humans. As all cannabis commercial formulations were first tested in preclinical studies, missing data on the pharmacokinetics of cannabinoids in humans may be available in animal models. Indeed, pharmacokinetics studies in pregnant and lactating women, in fetuses and in neonates are lacking; thus, animal data could be used to extrapolate the pharmacokinetics in these vulnerable populations. Therefore, it is important to assess which cannabinoids were tested, their concentration, their time to reach the maximal concentration (T_max_), their half-life (T_1/2_) and the sampling period for these types of population. Table 3 summarizes the pharmacokinetics parameters of cannabinoids in animals.

**TABLE 3:** Pharmacokinetics of cannabinoids in animal models (avant).

	Rats								Guinea pigs	
Measured Compounds	THC, 11-OH-THC, THC-COOH, CBD, CBDV, THCV and CBG								THC, CBD, 11-OH-THC, THC-COOH	
Matrices	Plasma						Urine	Maternal and fetal plasma	Plasma	
Mode of administration	Oral	Intravenous	Intranasal	Pulmonary	Subcutaneous	Intraperitoneal	Oral		Intravenous	Transdermal
Cmax (ng/ml)	39–3,200 (CBD) 5.5–3,200 (THC) 43 (THC-COOH) 20 (11-OH-THC) 2,200 (CBDV) 210 (THCV) 1,050 (CBG)	3,596–12,917 (CBD) 194–20,620 ng/ml (THC), 10 ng/ml (11-OH-THC), 6 ng/ml (THC-COOH)	19.9–35.4 (CBD) 49–66 (THC)	230–330 (CBD) 11–300 (THC) 3.2 (THC-COOH)	45–70 (CBD) 25–100 (THC) 5–10 (11-OH-THC) 11.9 (THC-COOH)	2,400–2,600 (CBD) 1,300 (CBDV) 400 (THCV) 810 (CBG)	8.5–9.3 (11-OH-THC) 18.4–22 (THC-COOH)	Maternal: 99–304 (THC) Fetal: 10–41 (THC)	269 (CBD) 197.7 (THC)	8.6–35.6 (CBD)
Tmax (hours)	1–8 (CBD) 0.7–6 (THC) 8 (THC-COOH) 1–2 (11-OH-THC) 0.5 (CBDV, CBG) 2 (THCV)		0.3–0.5 (CBD) 1.5–1.6 (THC)	0–15 min (CBD) 0–10 min (THC) 8 h (THC-COOH)	1 (CBD, THC, 11-OH-THC) 4 (THC-COOH)	0.5–2 (CBD) 4 (CBDV) 0.5 (THCV) 1 (CBG)		Maternal and fetal: 1 h (THC) (not Tmax)		31.2–38.4 (CBD)
Half-life (hours)	1.8–4.6 (CBD) 4.0 (CBDV) 1.5 (THCV) 1.7 (CBG)	1.1–1.4 (CBD) 1.3 (THC)	1.2–2.8 (CBD)	3.7 (THC)		7.8–10.1 (CBD) 6.7 (CBDV) 10 (THCV) 9.3 (CBG)			3.5 (CBD)	
Time of sampling	0–24 h after administration								0–48 h after administration	
Articles	Pryor et al. (1977); Hutchings et al. (1989); Valiveti and Stinchcomb, (2004); Valiveti et al. (2007); Paudel et al. (2010); Deiana et al. (2012); Zgair et al. (2015); Cherniakov et al. (2017b); Hlozek et al. (2017); Ravula et al. (2018); Fuchs et al. (2019); Izgelov et al. (2020a); Izgelov et al. (2020b); Izgelov et al. (2020c)								Blackard and Tennes, (1984); Izgelov et al. (2020b)	

	Dogs					Mice				
Measured Compounds	CBD, THC, 11-OH-THC					THC, 11-OH-THC, THC-COOH, CBD, CBDV, THCV, CBG				
Matrices	Plasma				Urine	Plasma				Maternal plasma, whole fetus body and amniotic fluid
Mode of administration	Oral	Trasndermal	Sublingual	Intravenous	Intravenous	Intraperitoneal	Oral	Intravenous	Subcutaneous	
Cmax (ng/ml)	346.3–845.5 (CBD) 3.6–77.1 (THC)	74.3–277.6 (THC)	18.5–24.5 (THC) 10.5–15.2 (CBD) 1.2–2.2 (11-OH-THC)	6,950–13,550 (CBD)	12 µg (CBD)	22.3–13,575 (THC) 2.0–39.3 (11-OH-THC) 8.3–506.3 (THC-COOH) 14,300 (CBD) 4,000 (CBDV) 880 (THCV) 40,800 (CBG)	129.5–2,200 (CBD) 470 (CBDV) 240 (THCV) 670 (CBG)	2,343.3 (CBD)	1.2–1.6 (THC) 22–34 (CBD) 2 (CBG)	Maternal plasma: 2,615.3 (CBD) Whole fetus body: 598.7 ng/g (CBD) Amniotic fluid: 74.4 (CBD)
Tmax (hours)	2–6 (CBD) 0.5–8 (THC)	10–12 (THC)	1–2 (THC, CBD, 11-OH-THC)			0–0.5 (THC) 0.25–1 (11-OH-THC) 0.5–1 (THC-COOH) 2 (CBD, CBG) 0.5 (CBDV, THCV)	1–2 (CBD) 0.5 (CBDV, THCV, CBG)			Amniotic fluid: 0.5 (CBD)
Half-life (hours)	1.6–3.3 (CBD) 0.8–3.5 (THC)			6.8–9.3 (CBD)	1.8 (CBD)	0.7–1.8 (THC) 1.2–3.5 (11-OH-THC) 2.7–4.2 (THC-COOH) 4.7 (CBD) 2.9 (CBG), 4.7(CBDV) 5.8 (THCV)		3.9 (CBD)		Maternal plasma: 4.9 Whole fetus bodies: 2.3 Amniotic fluid: 51.5
Time of sampling	0–96 h after administration					0–14 days after the administration				
Articles	Samara et al. (1988); Samara et al. (1990); Hasiwa et al. (2011); Bartner et al. (2018); Fernandez-Trapero et al. (2020)					Bornheim et al. (1995); Deiana et al. (2012); Elmes et al. (2019); Xu et al. (2019); Torrens et al. (2020); Uziel et al. (2020); Ochiai et al. (2021)				

	Monkeys				Pigs		Rabbits				
Measured Compounds	THC, 11-OH-THC, THC-COOH				THC		THC				
Matrices	Plasma			Maternal and fetal plasma	Plasma		Plasma				
Mode of administration	Intravenous	Oral	Intramuscular		Intravenous	Oral	Intravenous	Intranasal	Ophtalmic	Oral	Sublingual
Cmax (ng/ml)	5,000 (THC)	42–239 (THC) 3.9–44.7 (11-OH-THC) 3–53.5 (THC-COOH)	225–255 (THC)	Maternal: 1,419 (THC), 44 (THC-COOH) Fetal: 83 (THC) THC-COOH not detected	39.5–161 (THC)	0.17 (THC)	3–1,475 (THC)	20–31 (THC)	0.3–2.3 (THC)	0.9 (THC)	0.5–3.0 (THC)
Tmax (hours)		0.33 (THC) 1–2 (11-OH-THC, THC-COOH)	0.5–2.3 (THC)			12 (THC)		0.33–0.75 (THC)	0.3–7.5 (THC)	0.5 (THC)	3–4 (THC)
Half-life (hours)		2–2.7 (THC)			7.4–13.6 (THC)		1.1–66.2 (THC)				
Time of sampling	0–96 h after administration				0–48 h after administration		0–10 days after administration				
Articles	Perlin et al. (1985); Ginsburg et al. (2014); Ochiai et al. (2021)				Brunet et al. (2006); Itin et al. (2020)		Chiang et al. (1983); Leuschner et al. (1986); Mannila et al. (2004); Mannila et al. (2006); Al-Ghananeem et al. (2011)				

	Sheeps	Buffalos		Squirrel monkeys
Measured Compounds	THC	THC-COOH		THC
Matrices	Maternal and fetal plasma	Breast milk	Maternal and child urine	Maternal breast milk, urine and feces and child urine and feces
Mode of administration				
Cmax (ng/ml)		51 (THC-COOH)	Maternal urine: 17–289 (THC-COOH) Child urine: 8–25 (THC-COOH)	Breast milk: 0.2% of the maternal dose Maternal urine: 42% Maternal feces: 1% Child urine: 0.01% Child feces: 0.12%
Tmax (hours)	Maternal: 10 min Fetal: 90 min			
Half-life (hours)	Maternal and fetal: >10			
Time of sampling	0–24 h after administration			18 h after administration
Articles	Abrams et al.(1985)	Ahmad and Ahmad,(1990)		Chao et al.(1976)

#### 2.3.1 Pharmacokinetics of Cannabinoids in Non-Pregnant or Lactating Animals

Multiple preclinical models were used to assess the pharmacokinetics of cannabinoids: rats, mice, dogs, rhesus monkeys, guinea pigs, rabbits, and pigs (Pryor et al., 1977; Chiang et al., 1983; Perlin et al., 1985; Leuschner et al., 1986; Samara et al., 1988; Samara et al., 1990; Bornheim et al., 1995; Mannila et al., 2004; Valiveti and Stinchcomb, 2004; Brunet et al., 2006; Mannila et al., 2006; Valiveti et al., 2007; Paudel et al., 2010; Al-Ghananeem et al., 2011; Hasiwa et al., 2011; Deiana et al., 2012; Ginsburg et al., 2014; Zgair et al., 2015; Cherniakov et al., 2017b; Hlozek et al., 2017; Bartner et al., 2018; Ravula et al., 2018; Elmes et al., 2019; Fuchs et al., 2019; Xu et al., 2019; Łebkowska-Wieruszewska et al., 2019; Izgelov et al., 2020a; Izgelov et al., 2020b; Izgelov et al., 2020c; Fernández-Trapero et al., 2020; Itin et al., 2020; Torrens et al., 2020; Uziel et al., 2020; Michael, 2021). Pharmacokinetics parameters of THC, 11-OH-THC, THC-COOH, THCV, CBD, CBDV and CBG are presented in Table 3 after different modes of administration.

Torrens observed differences in the pharmacokinetics of THC, 11-OH-THC and THC-COOH between adolescent and adult mice suggesting an age-dependent pharmacokinetics of cannabinoids (Torrens et al., 2020). These results highlight the importance of studying the pharmacokinetics of cannabinoids in fetuses and newborns as their metabolism and their elimination could be significantly different. Also, variations in THC concentrations and T_max_ after oral consumption were observed depending on the fed/fasting statute of dogs suggesting that this parameter should be controlled in pharmacokinetics studies to avoid interindividual variability (Fernández-Trapero et al., 2020). Finally, Perlin and Ginsburg concluded that the pharmacokinetics of THC in rhesus monkeys is similar to humans. Therefore, data in this animal, especially in the pregnant and lactating model, should be used in priority to predict the pharmacokinetics of cannabinoids in humans (Perlin et al., 1985; Ginsburg et al., 2014).

Overall, pharmacokinetics profiles of cannabinoids in animals are consistent with that of humans and could be used to predict the transfer of cannabinoids to fetuses and neonates. In all animal models, except guinea pigs, THC, CBD, 11-OH-THC and THC-COOH were detected in plasma for at least 24 h. Therefore, these four cannabinoids should be considered for pharmacokinetics studies in animals.

#### 2.3.2 Pharmacokinetics of Cannabinoids in Pregnant and Lactating Animals

**FIGURE 2:**
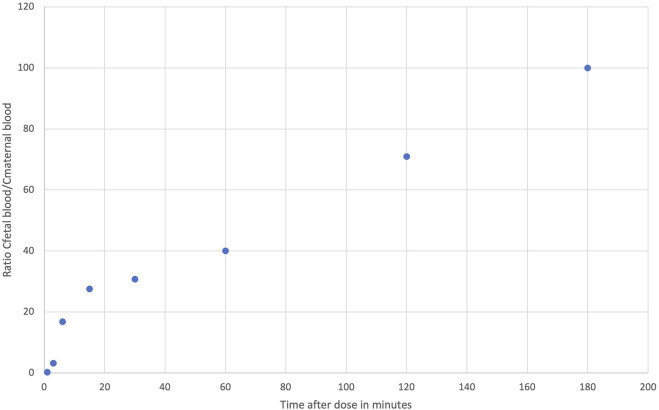
Ratios of fetal concentrations over maternal concentrations over 3 h in rhesus monkeys based on data obtained by Bailey et al. (1987).

Fetal THC concentrations in rats seem to be around 10% of the maternal concentrations 60 min after the administration of THC by gastric gavage, according to Hutchings et al. (Hutchings et al., 1989). Ochiai et al. assessed the pharmacokinetics of CBD in maternal plasma, fetuses (whole body) and amniotic fluid of mice over a 24-hour period after an intravenous injection of 10 mg/kg of CBD (Ochiai et al., 2021). The authors observed that CBD transfer to the fetus (whole body) began 15 min after CBD administration at a rate of 66.9%. THC and THC-COOH were measured in maternal and fetal plasma of rhesus monkeys after the intravenous administration of 0.3 mg/kg of THC by Bailey et al. (Bailey et al., 1987). If the THC-COOH metabolite was not detected in appreciable levels in fetal plasma after 3 h, THC concentration was found to peak at 3 min in maternal plasma and 15 min in fetal plasma suggesting a rapid transfer to the placenta. Ratios of THC concentrations in fetal plasma to THC concentrations in maternal plasma can be calculated over 3 h based on the data obtained by Bailey et al. and are shown in Figure 2. Three hours after THC administration, maternal and fetal THC plasma were equal, supposing a slower elimination of THC from fetal plasma than from maternal plasma showing worrisome data when compared to the study in rats aforementioned (Hutchings et al., 1989). Abrams assessed the transfer of THC in fetuses of sheep after the inhalation of marijuana cigarettes containing 3.19% of THC (Abrams et al., 1985). THC was first detected in maternal plasma at 3 min and peaked at 10 min whereas THC was first detected in fetal plasma at 10 min and peaked at 90 min. THC concentrations were always lower in fetal plasma than in maternal plasma (0–24 h). Data on THC concentrations in fetuses from different animal models are consistent. THC rapidly crosses the placenta barrier with a lag time of 10–15 min and has a slow elimination from the fetal compartment during the 3 h following peak concentrations.

THC and THC-COOH concentrations were also measured in breast milk of buffalos and squirrel monkeys (Chao et al., 1976; Ahmad and Ahmad, 1990). In buffalos, whose marijuana consumption accounts for approximately 5%–10% of their total vegetation consumption, THC-COOH was detectable in milk and urine specimens of both mother and offspring with concentrations in breast milk and offspring urine about 76 and 30% of maternal urine concentrations, respectively (Ahmad and Ahmad, 1990). Finally, Chao observed in breastfeeding infants of squirrel monkeys that 0.01% and 0.12% of the THC maternal dose were excreted in urine and feces, respectively, during the 18 h after breastfeeding (Chao et al., 1976). Moreover, approximately 0.2% of the labelled THC appeared in the milk. Limited interpretations can be made from these studies as neither the dose nor THC concentrations in breast milk over time are known. THC concentrations seems to be lower in breast milk than in maternal blood, and the exposition of offspring to cannabis appeared to be limited. No study measured THC concentrations in the blood of offspring.

## 3 Discussion

### 3.1 Lack of Knowledge on Cannabinoid's Pharmacokinetics

Preceding sections summarize research characterizing the exposure of animals and humans to cannabinoids. Extensive research has been published on the pharmacokinetics of THC and CBD in plasma of animals (rats and mice) and humans. However, only a handful of studies have looked at other cannabinoids of interests (CBD metabolites, THC-COOH glucuronide), either because of their activity or their long half-life, in vulnerable populations such as pregnant and lactating women and neonates. Table 4 summarizes the lack of data on the pharmacokinetics of cannabinoids identified by this review.

**TABLE 4:** Missing data on the pharmacokinetics of cannabinoids in animals, adults and newborns.

Model	Matrix	Missing data
Animals	Plasma, urine, feces, breast milk	• CBD metabolites and THC-COOH glucuronide concentrations
Adults	Plasma	• Cannabinoids concentrations in pregnant women
	Breast milk	• THC, 11-OH-THC, THC-COOH pharmacokinetics profile over 24 h and more • CBD metabolites concentrations • Pharmacokinetics of CBD and its metabolites after CBD only consumption • Effects of the mode of administration on cannabinoid concentration in breast milk
	Urine	• CBD metabolites concentrations
Newborns	Meconium	• CBD metabolites concentrations • Correlation between cannabinoid concentrations and the frequency of consumption • Correlation between cannabinoid concentrations and the last trimester of use during pregnancy
	Infant blood	• Cannabinoid concentrations

Data on the pharmacokinetics of cannabinoids during pregnancy and lactation are lacking. Recently the journal of Obstetrics and Gynecology has published guidelines on the use of cannabis during pregnancy and breastfeeding (Graves et al., 2022b). They concluded that more data are needed to guide clinical cares. Moreover, they pointed the urgency to make correlations between the pharmacokinetics and the pharmacodynamics of cannabis and to assess the pharmacokinetics and effects of CBD alone. The first step to understand the exposure of fetuses to cannabis during pregnancy will be to assess how the physiological changes during pregnancy will change the pharmacokinetics of cannabinoids in humans as it was never evaluated to date. Meconium has been the most tested matrix to assess the transfer of cannabinoids to fetuses. However, published studies using meconium samples were more qualitative than quantitative. Therefore, limited information can be obtained from meconium samples. Further studies should assess the ability of cannabinoids quantification in meconium to determine the last trimester of cannabis use and the frequency of consumption of women during pregnancy. Blood both in the umbilical cord and in the child will have the advantages of establishing the real exposure of the fetus to cannabis and to make correlation between the dose taken by the mother and this exposure. Future studies could use Dry Blood Spot (DBS) or Volumetric Absorptive Microsampling (VAMS) to collect infant blood as it is less invasive and more acceptable by parents (Lei and Prow, 2019). Finally, breast milk is the best matrix to assess the exposure of newborns to cannabis during breastfeeding. Unfortunately, data on the transfer of cannabinoids into breast milk is limited due to difficulties in recruiting lactating women using cannabis in clinical studies. New studies controlling the mode of administration of cannabis, the time of breast milk sampling and including CBD metabolites are critical.

In animals, THC, THC metabolites and CBD were well characterized in plasma of several models. As the popularity of CBD formulations increases, CBD metabolites should be included in animal studies that assess the safety of new CBD formulations. Other matrices than plasma have rarely been used in animal pharmacokinetics studies. Urine, feces, breast milk, amniotic fluid or even cord blood analysis could provide additional information on animal exposure to cannabinoids such as the elimination of cannabinoids, the length of exposure to cannabinoids and the transfer of cannabinoids to newborns during pregnancy and lactation. Indeed, only limited data is available on the exposure of newborns to cannabis during pregnancy and lactation. As cannabis is increasingly popular in these two populations, it is crucial to assess the exposure of newborns to cannabis during pregnancy and lactation and to assess the effects of cannabinoids on their development. As it is difficult to conduct this type of study in humans, animals could be a good alternative.

### 3.2 Prediction of Fetus and Neonate Exposure During Pregnancy and Lactation

**FIGURE 3:**
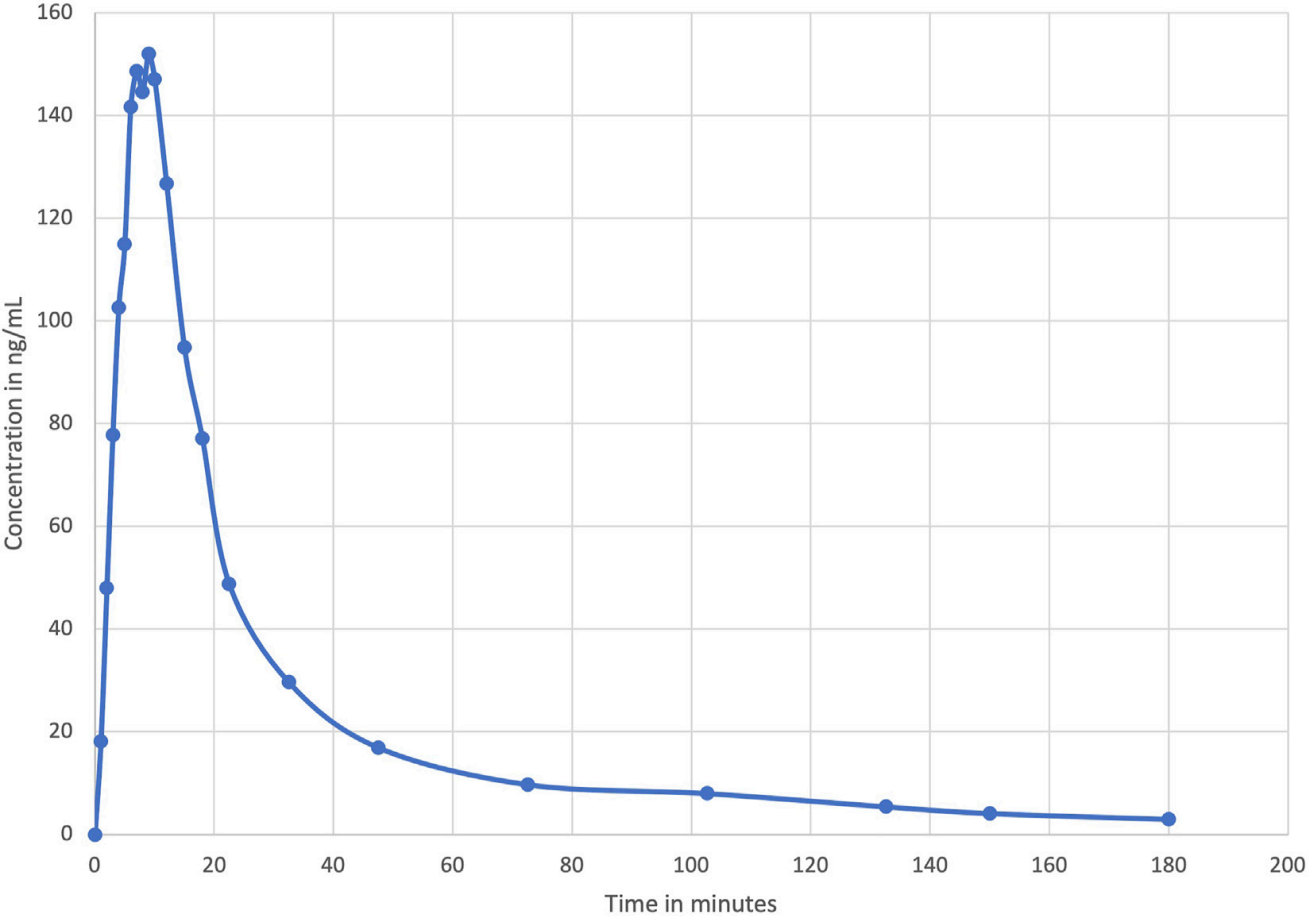
Mean plasma levels of THC during and after smoking a single 3.55% THC marijuana cigarette obtained by Huestis et al. (1992).

As mentioned previously, data on cannabinoid pharmacokinetics in pregnant and lactating women and neonates are scarce. While waiting for robust controlled studies, it is first important to understand and interpret the data obtained in the general population and in animals. These data could also be used to estimate, by extrapolation, the exposure of pregnant and lactating women and newborns to cannabinoids. We based our extrapolations on the concentrations obtained by Huestis et al., in 1992 (Huestis et al., 1992b). They measured THC, 11-OH-THC and THC-COOH concentrations in plasma samples of 6 volunteers with a history of marijuana use after the inhalation of cannabis in a single 3.55% THC marijuana cigarette (Figure 3). As a reminder, THC and 11-OH-THC are psychoactive while THC-COOH is a non-active metabolite.

**FIGURE 4:**
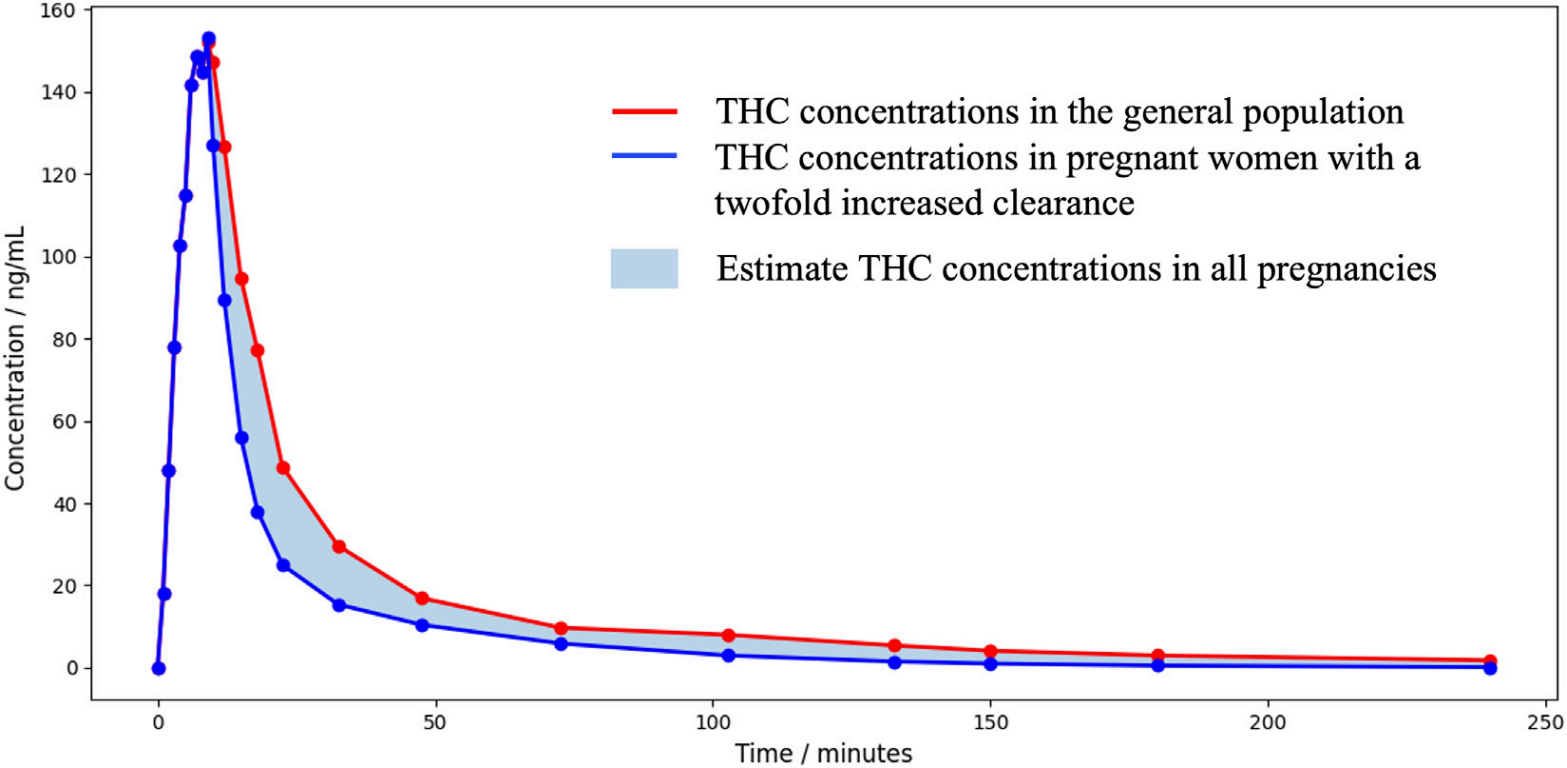
Area of theoretical THC concentrations in pregnant women over 3 h after a single 3.55% THC marijuana cigarette.

We previously stated that cannabinoids concentrations, volume of distribution and clearance can be altered during pregnancy. This alteration will be even more important as the pregnancy progresses. Grant et al. (2018) suggested that THC clearance could be increased by a factor of 2. Regarding the THC volume of distribution, no statement has been made, yet. However, studies that have assessed changes of this parameter for methadone, a drug with similar physicochemical properties as THC, observed no significant changes between pregnancy and post-partum period (Shiu and Ensom, 2012). Therefore, we decided to estimate cannabinoids concentrations in plasma of pregnant women considering the possibilities that either the THC volume of distribution and clearance are not affected by pregnancy or that the THC volume of distribution is unchanged, and clearance is affected by a factor of 2. The theoretical Figure 4 shows an area of calculated THC concentrations in pregnant women.

**FIGURE 5:**
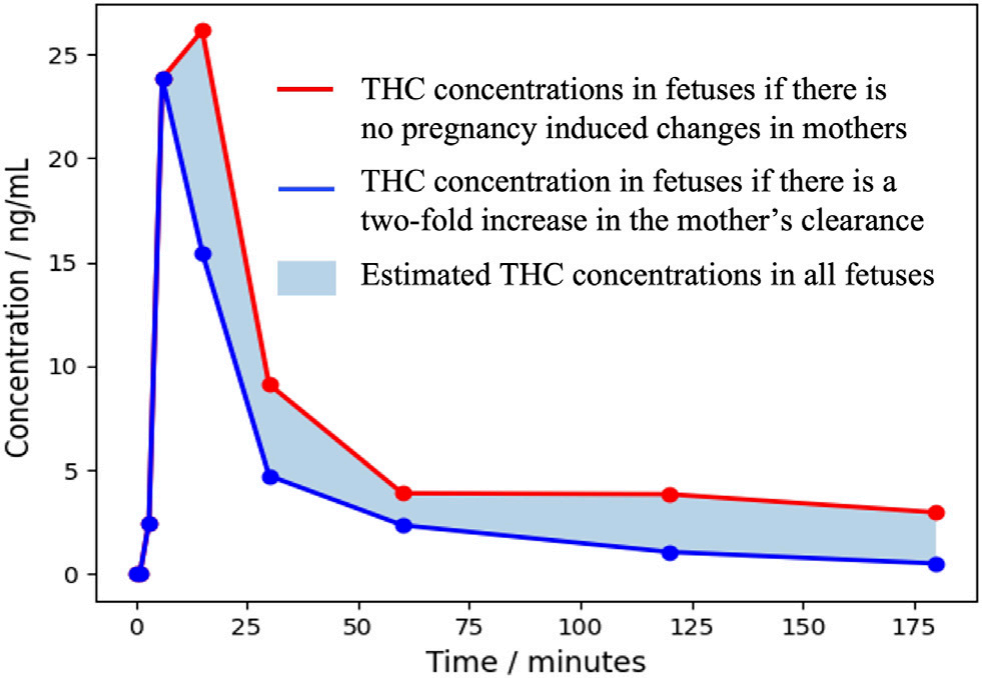
Area of theoretical THC concentrations in fetuses over 3 h after a single 3.55% THC marijuana cigarette.

Based on this information, a pregnant woman could be exposed to similar or lower THC concentrations and similar or higher metabolites concentrations. The psychoactive effect might be similar or shorter. In fetuses, it is already well known that THC, CBD and CBN cross the placental barrier as they were detected in meconium. Metabolites concentrations measured in meconium could be the result of their passage or the transformation by the fetus metabolism. To predict the exposure of fetuses to THC, we multiplied the ratio of THC concentrations in rhesus monkey fetal plasma to THC concentrations in maternal plasma presented in Figure 2 by the calculated THC concentrations in pregnant women from Figure 4, as the pharmacokinetics of THC in rhesus monkeys was proven to be similar to that of humans. The theoretical Figure 5 represents the area of calculated THC concentrations in fetal plasma. Bailey et al. (1987) observed no appreciable THC-COOH concentrations in fetal plasma of rhesus monkeys when maternal THC-COOH concentrations were similar to that of Huestis et al. suggesting both that THC-COOH do not cross the placenta and that the monkey fetus does not metabolize THC at an appreciable level at this stage of development (around 90% of the pregnancy). These results suggest that human fetuses will be exposed to significant THC concentrations that will be eliminated much more slowly than in adults. Indeed, according to Figure 2, ratios of THC concentrations in rhesus monkey fetal plasma to THC concentrations in maternal plasma are increasing with time suggesting a slower elimination of THC by the fetus. This can be explained by the limited fetal elimination mechanisms (Morgan, 1997). Therefore, depending on the frequency of cannabis use by the mother, a fetus could be continually exposed to appreciable levels of THC during the pregnancy as it is taking several days for an adult to eliminate THC. Moreover, as a lipophilic drug, THC could easily transfer to the fetus brain and accumulate. THC-COOH was detected in human meconium and umbilical cord suggesting that this metabolite crosses the placental barrier and/or is produced by the metabolism of the fetus. However, THC-COOH is a non-active metabolite. Even though 11-OH-THC was never tested in pregnant animal models, it is unlikely that fetuses will be exposed to significant levels because of its higher polarity and its low levels in adults. Finally, it is possible that other active cannabinoids cross the placental barrier and the blood brain barrier and accumulate similarly to THC. Caution should be taken with these predictions as it is based on cannabinoid concentrations after a single dose of THC. Indeed, it is likely that after multiple cannabis doses, THC concentrations will be higher and eliminated more slowly.

From the studies published on LactMed regarding the transfer of cannabinoids in breast milk, we can conclude that THC, 11-OH-THC, THC-COOH, CBD and CBG are present in breast milk for at least 40 h (Baker et al., 2018; Bertrand et al., 2018; Moss et al., 2021; Wymore et al., 2021). THC could be detectable in breast milk for up to 6 days with higher concentrations than in plasma. The authors estimated that an infant would be exposed to a daily dose between 1.4 and 8 mcg/kg. Although these values appeared to be low, it was demonstrated by Perez-Reyes that THC from breast milk is absorbed and metabolized by the infant (Presence of Δ9, 1982). Indeed, the absorption of a drug like THC in neonates is likely to be higher than in adults due to the immaturity of the enzymatic systems. Moreover, it is possible that the quantity of THC absorbed by the neonates will be eliminated more slowly due to the low expression of CYP450 during the first months after delivery and will transfer to its brain due to its high lipophilicity (O'Hara et al., 2015).

As stated in the introduction, the use of cannabis during pregnancy and breastfeeding can induce negative birth outcomes such as reduced birth weight and increased risk of prematurity, behavioral and neurocognitive impairment, and cognitive deficits in children (Fried and Watkinson, 1990; Day et al., 1994; Goldschmidt et al., 2004; Smith et al., 2006; Smith et al., 2010; Hayatbakhsh et al., 2012; Conner et al., 2016; Gunn et al., 2016; Smith et al., 2016; Porath, 2018; Paul et al., 2021). Lately, Paul et al. observed in 11,489 children that prenatal exposure to cannabis was associated with a greater risk of psychopathologies than no exposure (Paul et al., 2021). Additionally, they found a higher risk of psychopathologies in children from mothers who continued their cannabis consumption throughout pregnancy suggesting that prolonged cannabis exposure has a direct impact on brain development.

## 4 Conclusion

Data on the pharmacokinetics of cannabinoids are still limited for many cannabinoids and especially for vulnerable populations such as pregnant and lactating women and neonates. With worrisome data on the exposure and the potential effects of cannabis on fetuses and neonates, scientific research should focus on filling this knowledge gap as it is a growing societal matter. Indeed, cannabis use during pregnancy and lactation has risen over the years, and with the recent legalization, it is more than ever important to assess cannabis transfer in the fetus and the breastfed child and to update official recommendations in that regard. This review will help to guide future clinical studies aimed at monitoring fetal and neonatal toxicity and assessing correlation between the pharmacokinetics and the pharmacodynamics of cannabis.
